# Super Responders in Moderate-to-Severe Atopic Dermatitis Under Treatment with Dupilumab: An Explorative Real-World Study

**DOI:** 10.3390/jcm14103480

**Published:** 2025-05-16

**Authors:** Luca Mastorino, Pedro Mendes-Bastos, Giovanni Cavaliere, Niccolo Siliquini, Michela Ortoncelli, Pietro Quaglino, Simone Ribero

**Affiliations:** 1 Dermatologic Clinic, Department of Medical Sciences, University of Turin, 10121 Torino, Italy; gcavaliere@cittadellasalute.to.it (G.C.); nicsiliquini@gmail.com (N.S.); mortoncelli@cittadellasalute.to.it (M.O.); pietro.quaglino@unito.it (P.Q.); simone.ribero@unito.it (S.R.); 2 Dermatology Center, Hospital CUF Descobertas, 1998-018 Lisboa, Portugal

**Keywords:** early responders, super responders, atopic dermatitis, dupilumab, treat-to-target, EASI

## Abstract

**Background:** A shared definition of therapeutic targets in the treatment of atopic dermatitis (AD) allows for the identification of patients who respond rapidly (early responders [ERs]) and optimally (super responders [SRs]) to systemic treatments. A concomitant achievement of EASI75/≤7, PP-NRS ≤ 4, SCORAD-75/≤24, POEM ≤ 7, and DLQI ≤ 5 at 6 months of treatment has been defined as an ideal target for AD. **Methods:** Patients aged ≥ 12 years treated with dupilumab for moderate-to-severe AD in an Italian center for at least 2 years were analyzed. We defined ERs as those who achieved EASI ≤ 7, PP-NRS ≤ 4, POEM ≤ 7, and DLQI ≤ 5 within 32 weeks, and SRs and long responders (LRs) as those who maintained the target at 1 year and at 2 years, respectively. We subsequently compared baseline characteristics between those who fell within the above definitions and those who did not. **Results**: Of 171 patients with AD, 76.6% were ERs, 49.1% SRs, and 40.4% LRs. Achievement of combined outcomes was shown by 37.11% of patients at 16 weeks, and increased at the following time points by more than half of patients at 2 years of treatment. Except for a high baseline POEM that appears to be unfavorable for achieving early response (OR 0.93, CI 0.88–0.98, *p* = 0.006), no baseline characteristics were associated with ERs, SRs, or LRs in this population. **Conclusions:** According to our definition of responders, we were unable to identify a patient profile at baseline that predicts optimal therapeutic outcomes with dupilumab. Only baseline POEM seems to affect achievement of the selected outcomes. Dupilumab showed a rapid achievement of the outcomes with a stable response after 4 months of treatment, according our definitions. Shared definitions of the different categories of patient responders and a common therapeutic target are necessary for optimal management of AD.

## 1. Introduction

Atopic dermatitis (AD) is one of the most common inflammatory skin diseases. The disease is characterized by a chronic and relapsing course with flare-ups of itchy eczematous lesions and dry skin. The prevalence of AD has increased on most continents and has stabilized in Europe and North America, affecting 15–20% of children and 1–3% of adults [1,2,3].

The pathophysiology of AD is complex and multifactorial and includes genetic disorders, epidermal barrier defects, alterations in the immune response, and environmental alterations that cause skin barrier abnormalities and immune dysfunction that are considered crucial [4,5].

Several treatments are currently available, and their use depends on disease severity. In cases of mild-to-moderate AD, the treatment is based on topical calcineurin inhibitors or topical corticosteroids, with phototherapy as an adjunct. In moderate-to-severe AD requiring systemic treatment, the only traditional on-label long-term treatment is cyclosporine [6,7]. Several treatment options have recently become available, including the biological drugs dupilumab, tralokinumab, and lebrikizumab, which are currently available and recommended for the treatment of moderate-to-severe AD, along with the Janus kinase inhibitors (JAKis) abrocitinib, baricitinib, and upadacitinib [8].

The introduction of advanced therapies such as biological drugs and small molecules has elevated the therapeutic targets in managing patients with AD [9]. Dupilumab, the first biologic agent approved by the Food and Drug Administration in 2020 for the treatment of pediatric patients aged ≥ 6 with moderate-to-severe AD, targets the IL-4Rα subunit and inhibits inflammatory responses by blocking the signaling of IL-4 and IL-13. Tralokinumab and nemolizumab are also biologics that inhibit inflammatory responses by blocking interleukins. Additionally, upadacitinib, baricitinib, abrocitinib, and delgocitinib are JAKis that effectively block the JAK/STAT pathway, leading to inhibition of interleukin signaling [10].

In clinical trials, treatment targets are based on scoring systems for disease severity, as recommended by the Harmonizing Outcome Measure for Eczema (HOME) initiative, with the primary endpoint being a reduction of at least 75% of the baseline Eczema Area and Severity Index (EASI) score (EASI75) [9]. The question, however, is if these are useful targets in real-world settings and how this goal should be achieved in everyday clinical practice. To apply a treat-to-target strategy that is meaningful for both the patient and physician, validated tools for measuring the effects of treatment should be included in the decision-making process when choosing which targets should be considered as treatment success [9].

Two studies based on Delphi consensus by Vestergard et al. and De Bruin Weller et al. identified EASI75 or absolute EASI ≤ 7, SCORAD (Scoring AD) 75, or an absolute SCORAD of 24 or less as criteria for therapeutic success at 6 months in a treat-to-target strategy. In addition, the patient should achieve specific patient-reported outcomes (PROs): PP-NRS (Peak Pruritus Numerical Rating Scale) ≤ 4, a DLQI (Dermatology Life Quality Index) ≤ 5, and a POEM (Patient-Oriented Eczema Measure) ≤ 7. According to the authors, a patient who achieves all the identified criteria simultaneously can be considered a “responder” to treatment in clinical practice [9,11]. The targets identified by the authors appear ambitious in clinical practice even with current biological drugs and would oblige the clinician to use numerous scores and questionnaires. In particular, using SCORAD, which synthesizes both the objective evaluation reflected by EASI and the symptom data (itch), appears redundant. It could either be excluded or replace both EASI and PP-NRS; however, EASI and PP-NRS are far more commonly used in real-world practice and clinical trials. Silverberg et al., in their analysis of patients treated with dupilumab, proposed that achieving EASI50, a 3-point reduction in itch, and a 4-point improvement in DLQI from week 24 to 52 could be considered sufficient therapeutic targets [12].

Identifying the correct therapeutic target also allows for the identification of patients who will respond more quickly than others. Borrowing terminology from studies on psoriasis, these patients are referred to as “super responders” [13]. Ruiz-Villaverde et al. highlighted the lack of consensus on this definition and concluded that the outcomes and timing of achievement should be tailored to the nature of the disease. For AD, they proposed to consider not only clinical outcomes like EASI but also PROs such as DLQI and PP-NRS, and suggested 52 weeks as an appropriate time point for evaluation [14]. Due to the lack of consensus on the definition of a “super responder” (SR) and the relatively recent introduction of target criteria in managing AD, the present study proposes explorative definitions for early responders (ERs), SRs, and “long responders” (LRs). We applied these explorative concepts to our population of patients with AD who were treated with dupilumab.

## 2. Materials and Methods

### 2.1. Population

All patients with moderate-to-severe AD and treated with dupilumab (given at the approved dose) aged ≥ 12 years at the Dermatologic Clinic of the University of Turin, Italy from December 2018 to March 2024 were enrolled. The present study was approved by our Institutional Review Board (Comitato Etico Territoriale Interaziendale AOU Città della Salute e della Scienza di Torino) under the protocol SS-DERMO-13, and all patients gave written informed consent.

### 2.2. Exclusion Criteria

- Age ≤ 12 years;
- Dupilumab administration for less than 2 years;
- Clinical follow-up less than 2 years;
- Missing clinical data at 16 or 32 weeks of treatment, or at 1 and 2 years of treatment.

### 2.3. Outcomes

Baseline characteristics, personal and family history, and previous treatments were collected.

The following were considered as outcomes:

- Absolute mean value of EASI, PP-NRS, DLQI and POEM at each time point;
- Achievement of EASI ≤ 7, PP-NRS ≤ 4, DLQI ≤ 5, and POEM ≤ 7 at each time-point.

The following time points were considered: baseline (T0), 16 weeks (W) (T1), 32 weeks (T2), 48 weeks (1 year) (T3), 64 weeks (T4), 80 weeks (T5), and 96 weeks (2 years) (T6).

### 2.4. Definitions

- Responder (Re): achievement of treatment targets EASI ≤ 7, plus PP-NRS ≤ 4. DLQI ≤ 5, and POEM ≤ 7.
- ER: achievement of therapeutic targets (see above) at 16 weeks of treatment (T1) or 32 weeks of treatment (T2).
- SR: ER who maintained treatment targets at 1 year (T3).
- LR: SR who maintained treatment targets at 2 years (T6).

### 2.5. Endpoints

(i) Description of the trend of outcomes analyzed, patient Re status, and number of patients who were ERs, SRs, or LRs.
(ii) Identification of baseline and clinical characteristics possibly related to being ERs, SRs, or LRs.

### 2.6. Statistical Analysis

Descriptive statistics were used to evaluate the data set according to the number of patients and their proportion in the groups related to the categorical variables; mean and standard deviation (SD) and median (Q1–Q3) were used for continuous variables, whether normally distributed or not.

Univariate linear regression, followed by a mixed-effects logistic regression model (including all analyzed factors with *p* < 0.20 at linear analysis), was employed to investigate potential factors affecting ER, SR, and LR status. A *p* value < 0.05 was considered significant. The analyses were conducted using STATA 15.1 SE (StataCorp., 2017, College Station, TX, USA); all tests were two-sided.

## 3. Results

Out of 456 patients with dupilumab in our clinic with retrievable data, 171 met the inclusion criteria and were enrolled in the study. The mean age was 41.13 years (SD 16.52), with 55.4% of participants being male. The main baseline characteristics are shown in Table 1.

**Figure 1:**
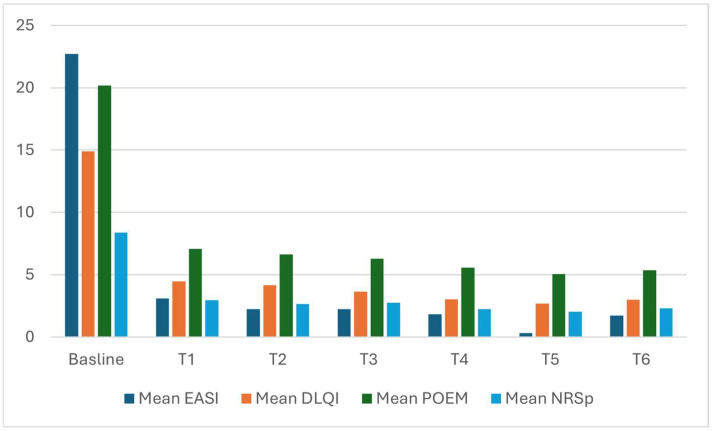
Trend from baseline to 2 years of mean and median EASI, DLQI, POEM, NRSp. EASI (eczema area severity index), DLQI (dermatology life quality index), POEM (patient-oriented eczema measure), NRSp (numerical rating scale pruritus).

The mean EASI decreased from 22.73 (SD 11.1) at baseline to 3.09 (SD 4.07) at 16 weeks, and then further reduced to 1.73 (SD 2.38) after 2 years of treatment (T6). The mean DLQI decreased from 14.9 (SD 7.1) at baseline to 2.98 (SD 4.34) at T6. Similar responses were observed for mean POEM and mean PP-NRS, with reductions from 20.18 (7.1) to 5.35 (SD 5.35), and from 8.36 (SD 1.88) to 2.29 (SD 2.37), respectively, from baseline to T6 (Table 2, Figure 1).

**Figure 2:**
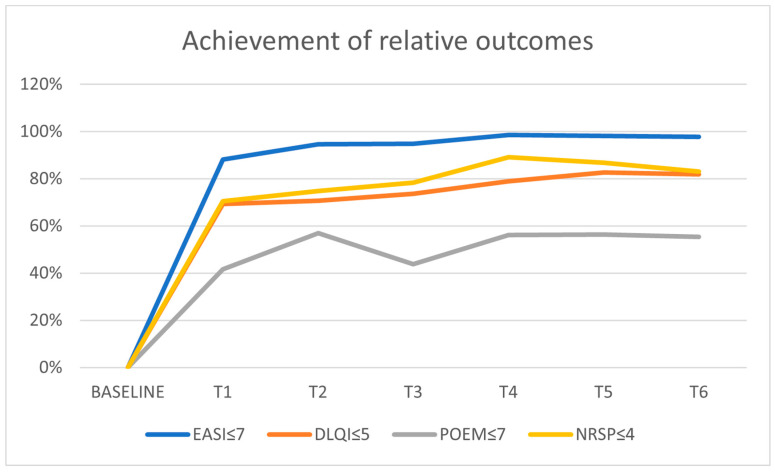
Achievement of POEM ≤ 7, DLQI ≤ 5, NRSp ≤ 4, and EASI ≤ 7 at baseline, 16, 32, 48, 64, 80, and 96. weeks (2 years). EASI (eczema area severity index), DLQI (dermatology life quality index), POEM (patient-oriented eczema measure), NRSp (numerical rating scale pruritus), w (weeks).

Regarding treatment targets, EASI ≤ 7 was achieved by 88.2% of patients at 16 weeks and remained stable between 95% and 100% for up to 2 years of therapy. DLQI ≤ 5 was met by 69.4% of patients at the first measurement and by nearly 82% after 2 years of treatment. POEM ≤ 7 improved steadily and remained stable, with 73.1% of patients reaching this target at T5 and 72.3% achieving it after 2 years. The highest rate of PP-NRS was observed at T4, with almost 90% of patients meeting the target, but this declined to 83% after 2 years (Table 2, Figure 2).

**Figure 3:**
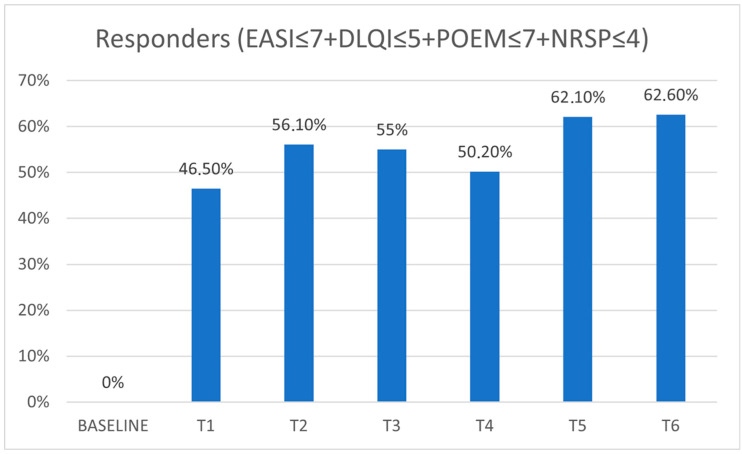
Percentage of patients achieving POEM ≤ 7, DLQI ≤ 5, NRSp ≤ 4, and EASI ≤ 7 together (i.e., “Responders”). EASI (eczema area severity index), DLQI (dermatology life quality index), POEM (patient-oriented eczema measure), NRSp (numerical rating scale pruritus).

Following the previously discussed treat-to-target strategy, the percentage of patients achieving Re status ranged from 46.5% at 16 weeks to 62.6% at 2 years of therapy, which were the time points with the highest outcomes (Table 2 and Figure 3). Between T1 and T2, 76.6% of patients reached the treatment targets, resulting in ER classification. Additionally, 49.1% of patients were classified as SRs, while 40.4% achieved and maintained the targets for up to 2 years of therapy (LR) (Table 2).

Patients classified as ERs tended to have a lower mean baseline POEM score compared to non-ER patients, with scores of 19.53 vs. 22.2. Multivariate analysis showed that as the baseline POEM increased, the likelihood of being an ER decreased (OR 0.93, CI 0.88–0.98, *p* = 0.006). No baseline characteristics were significantly associated with being an SR. However, this group had a higher proportion of males (60.7 vs. 50.5%) compared to non-SR patients, and less history of using calcineurin inhibitors and systemic steroids. Similarly, for LRs, no significant correlations were found with baseline characteristics, although this group also showed a trend towards less previous use of topical immunomodulators and systemic steroids (Table 3).

## 4. Discussion

To our knowledge, this is the first study deploying the concept of SRs and their surrogates (LRs and ERs) as outcomes of response. We observed that the achievement of all outcomes analyzed was present as early as 16 weeks and tended to stabilize at later time points. Overall, the achievement of a therapeutic response, as defined by Re, remained stable, with a slight increase from 46.5% at T1 to 62.6% at T6. It is noteworthy that nearly all patients achieved the clinical efficacy outcome of EASI ≤ 7, with values ranging from 88% to 98% at different time points. In contrast, the improvement in PROs was more limited, particularly for POEM ≤ 7, which ranged from 58% to 72%, largely justifying the overall data on Re patients. Three-quarters of patients reached the therapeutic target between weeks 16 and 32, which we defined as ERs. However, maintaining these targets in the same individuals was less successful, with only 50% achieving them at 1 year (SR) and 40% at 2 years (LRs). Aside from the mean baseline POEM score, which predicted target attainment between 16 and 32 weeks, no other baseline characteristics were able to predict ER, SR, or LR status, suggesting that the therapeutic response to dupilumab is largely independent of clinical features at baseline.

The definition of an SR for AD was first established in the Dutch registry, published by Ariëns et al. This group identified three key domains to categorize a patient as an SR: improvement in signs (EASI score), symptoms (PP-NRS), and QoL (DLQI score) [15]. Nettis et al. subsequently adopted this term for these specific targets, requiring EASI 75, a 4-point reduction in PP-NRS, and a 4-point reduction in DLQI by week 16 of dupilumab treatment to be considered as an SR or late SR [16]. An Italian study defined early SRs as those who met the targets at 4 weeks [15,16]. Significant differences were noted in target achievement at 16 weeks, with 40% in the Dutch study and 66% in the Italian study [15,16]. Nettis et al. found that 25.3% were SRs, and factors associated with being an early or late SR included the presence of allergic conjunctivitis, asthma, younger mean age, lower baseline PP-NRS, and early onset of disease [16]. Alegre-Bailo et al. defined an SR to tralokinumab as achieving EASI75 within 12 weeks. Albeit small (n *=* 9 patients), their study showed that SRs were significantly younger (44.2 vs. 63.3 years old), but no significant differences were found regarding previous systemic treatments or response to cyclosporine A [17].

Our findings align with recent reports by Limao et al. who found no significant differences in baseline clinical characteristics or biomarkers among SRs (defined similarly to our ER). They identified a 26.7% rate of SRs at 6 months, which is lower than our ER rate of 76.6% [18].

Two years after the publication by Ruiz-Villaverde et al., a clear definition of an SR in AD remains elusive, with different authors adopting various definitions [14]. Unlike psoriasis, where the definition of an SR has been promoted through the GUIDE study, there is no similar ongoing randomized control trial for AD that has ensured broad agreement on selection criteria [19]. The term SR may seem unnecessary, with some authors preferring terms like “clinically-meaningful response” to assess treatment adequacy [20]. Furthermore, real-world data have not precisely identified the baseline characteristics of patients who are SRs and often overlook long-term response, which is crucial, given the numerous therapeutic options available [14]. A progressive refinement of criteria for targets of response might lead to a more consistent therapeutic strategy over time [9,11]. Currently, with biologic drugs, these targets remain ambitious, and long-term maintenance is still limited among patients on treatment. A separate discussion should address small-molecule drugs, where overall targets such as EASI 75, EASI ≤ 7, PP-NRS ≤ 4, POEM ≤ 7, and DLQI ≤ 5 at 6 months might be more achievable. Real-life comparisons of these targets across different drug classes, including potential side effects and long-term effectiveness, are needed.

The limitations of our study include the typical constraints of retrospective studies. The number of patients analyzed was relatively small compared to the total number treated with dupilumab at our center. The inclusion criteria, aimed at minimizing missing data, also reduced the number of eligible patients, particularly due to the COVID-19 pandemic, which necessitated telephone follow-ups without clinical assessments and collection of PROs. Additionally, only patients with a follow-up up to 2 years were included, excluding those who discontinued treatment earlier, which could inherently select “good responders”. Our definitions of ER, SR, and LR are original, explorative, and based on the latest therapeutic targets, and should be validated by further research and consensus based definitions.

The recent definition of “minimal disease activity” could lead to better targeting of SR according to the new response targets in patients with AD, rendering the previously discussed targets outdated. In any case, real-life applications of this new outcome are still scarce [21].

## 5. Conclusions

A shared definition of therapeutic targets in the treatment of AD can help to identify the so-called SR who might benefit from advanced therapeutic options. It is crucial to include PROs alongside clinical criteria when defining therapeutic targets and SRs. Attention must be given not only to short-term responses, but also to long-term outcomes with new therapies. According to our definition of responders, we were unable to identify a patient profile at baseline that would predict optimal therapeutic outcomes with dupilumab. Only baseline POEM seems to affect achievement of the selected outcomes. Dupilumab showed a rapid achievement of the outcomes with a stable response after 4 months of treatment, according to our definitions. Further research, including studies with other biologics and small molecules, is needed to better understand the practicality and effectiveness of treat-to-target strategies in AD and to improve the identification of ERs, SRs, and LRs.

## Figures and Tables

**Table 1:** Baseline demographic characteristics and medical history.

Patients, N	171
Mean age (SD)	41.13 (16.52)
Mean BMI (SD)	23.85 (3.99)
Mean age of onset (SD)	14.09 (19.98)
Sex (male) %	55.6%
Smoking habit %	
no	56%
ex	11.9%
yes	6.6%
Alcohol (%)	
No	42.3%
Sometimes	51.2%
Yes	6.6%
Pediatric onset (%)	63.9%
Prurigo-like lesions (%)	7%
Family history (%)	37%
Predisposition to allergic conjunctivitis (%)	24.4%
Previous topical steroids (%)	98.8%
Previous topical calcineurin inhibitor (%)	45.9%
Previous phototerapy (%)	8.8%
Previous systemic steroids (%)	95.3%
Previous cyclosporine (%)	82.9%
Mean IgE T0 (SD)	4170.2 (6149.73)

**Table 2:** Trend from baseline to 2 years of mean and median EASI, DLQI, POEM, NRSp. Achievement of POEM ≤ 7, DLQI ≤ 5, NRSp ≤ 4, and EASI ≤ 7 at baseline, 16, 32, 48, 64, 80, and 96 weeks (2 years). “Responders” at each time point. Number of ERs, SRs, and LRs according to our definition. N (number), SD (standard deviation), Q (quartile), EASI (eczema area severity index), DLQI (dermatology life quality index), POEM (patient-oriented eczema measure), NRSp (numerical rating scale pruritus), w (weeks) Re (responder), ER (early responder), SR (super responder), LR (long responder).

	Base Line	T1	T2	T3	T4	T5	T6
Mean EASI (SD)	22.73 (11.1)	3.09 (4.07)	2.23 (2.73)	2.22 (2.73)	1.81 (1.98)	0.3 (0.8)	1.73 (2.38)
Median (Q1–Q3)	24 (15.3–28)	2 (0–4)	1 (0–3)	1.35 (0–3)	1 (0–3)	0 (0–0)	1 (0–3)
Mean DLQI (SD)	14.9 (7.1)	4.45 (4.94)	4.15 (5.2)	3.64 (4.36)	3.04 (3.9)	2.69 (3.16)	2.98 (4.34)
Median (Q1–Q3)	15 (10–20)	3 (1–6)	2 (1–6.25)	2 (1–6)	1 (0–5)	1 (0–4)	1 (0–4)
Mean POEM (SD)	20.18 (7.1)	7.08 (5.7)	6.61 (6.12)	6.29 (5.57)	5.58 (5.46)	5.04 (5.08)	5.35 (5.66)
Median (Q1–Q3)	22 (16–26)	6 (2.25–10.75)	5 (2–10)	5 (2–9)	4 (1–8)	4 (1–8)	4 (1–8)
Mean NRSp (SD)	8.36 (1.88)	2.96 (2.58)	2.66 (2.4)	2.74 (2.37)	2.25 (2.3)	2.04 (2.04)	2.29(2.37)
Median (Q1–Q3)	9 (7–19)	2 (1–5)	2 (1–4.5)	2 (1–4)	2 (0–3)	2 (0–3)	2 (0–3)
EASI ≤ 7	0%	88.2%	94.6%	94.7%	98.6%	98.2%	97.7%
DLQI ≤ 5	0%	69.4%	70.7%	73.7%	79%	82.6%	81.9%
POEM ≤ 7	0%	57.8%	66.5%	66.7%	69.7%	73.1%	72.3%
NRSP ≤ 4	0%	70.4%	74.9%	78.4%	89.1%	86.8%	83%
Re	0%	46.5%	56.1%	55%	50.2%	62.1%	62.6%
ER		76.6%					
SR				49.1%			
LR							40.4%

**Table 3:** Comparison of baseline demographic characteristics and medical history between patients: ER and non-ER, SR and non-SR, LR and non-LR. A *p* value < 0.05 was considered significant. Significant *p* values are indicated in bold. N (number), SD (standard deviation), OR (odds ratio), M (male), EASI (eczema area severity index), DLQI (dermatology life quality index), POEM (patient-oriented eczema measure), NRSp (numerical rating scale pruritus), ER (early responder), SR (super responder), LR (long responder), *p* (*p*-value).

	ER	Non-ER	*p*-Value	Multivariate Analysis (ER)	SR	Non-SR	*p*-Value	Multivariate analysis (SR)	LR	Non-LR	*p*-Value	Multivariate Analysis (LR)
Mean Age (SD)	41.75 (16.22)	39.13 (17.51)	0.381		41.75 (16.29)	40.54 (16.61)	0.633		41.64 (15.79)	40.79 (17.06)	0.744	
Mean BMI (SD)	24.1 (3.92)	23.04 (4.18)	0.149	OR 1.04 (0.96–1.14), *p* = 0.474	24.04 (4.02)	23.67 (3.98)	0.549		24 (4.02)	23.75 (3.99)	0.687	
Age of onset: Mean (SD)	13.18 (19.22)	17.08 (22.26)	0.282		13.95 (20.08)	14.23 (20)	0.928		13.81 (20.01)	14.29 (20.06)	0.879	
Sex (M) %	56.5%	52.5%	0.657		60.7%	50.5%	0.182	OR 0.65 (0.31–1.37), *p* = 0.255)	59.4%	52.9%	0.403	
Smoking habits %			0.322				0.783				0.627	
no	54.3%	61.6%			58.1%	53.7%			59%	51.5%		
ex	13.9%	5.1%			10.5%	13.4%			11%	13.2%		
yes	31.8%	33.3			31.4%	32.9%			30%	35.3%		
Alcohol %			0.556				0.692				0.629	
No	40.3%	48.7%			42.7%	41.9%			43.5%	41.4%		
Sometimes	53.5%	43.6%			52.4%	50%			52.2%	50.5%		
Yes	6.2%	7.7%			4.9%	8.1%			4.3%	8.1%		
Pediatric onset %	65.9%	57.5%	0.334		63.1%	64.7%	0.827		62.3%	65%	0.721	
Prurigo-like lesions %	8.2%	3.1%	0.295		8.5%	7.1%	0.936		8.3%	6.2%	0.447	
Familiarity %	39.7%	28.2%	0.194	OR 0.89 (0.45–1.76), *p* = 0.735	24.4%	24.4%	0.285		39.8%	32.8%	0.363	
Predisposition to allergic conjunctivitis %	24.2%	25%	0.92		24.4%	24.4%	0.997		23.8%	25.4%	0.812	
Previous topical steroids %	99.2%	97.5%	0.416		100%	97.7%	0.497		100%	98%	0.517	
Previous topical calcineurin inhibitors %	43.8%	52.5%	0.337		38.6%	52.9%	0.061	OR 1.47 (0.71–3.01), *p* = 0.297	38.2%	51%	0.102	OR 1.25 (0.66–2.39), *p* = 0.497
Previous phototerapy %	9.2%	7.5%	0.512		9.6%	8%	0.791		11.8%	6.9%	0.283	
Previous systemic steroids %	94.6%	97.5%	0.889		92.8%	97.7%	0.161	OR 1.69 (0.26–10.81), *p* = 0.581	92.6%	97.1%	0.169	OR 2,81 (0.63–12.41), *p* = 0.174
Previous cyclosporine %	81.5%	87.5%	0.87		80.7%	85.1%	0.453		79.4%	85.3%	0.318	
Mean IgE (SD) T0	4481.56 (7316.1)	4412.02 (5698.9)	0.961		3434.22 (4605.5)	4992.06 (7434.5)	0.155	OR 1 (1–1), *p* = 0.326	3881.86 (5708.75)	4629.2 (6829.75)	0.507	
Mean EASI (SD) T0	22.58 (11.26)	23.23 (10.61)	0.561		22.81 (11.69)	22.66 (10.54)	0.645		22.49 (11.53)	22.89 (10.83)	0.718	
Mean DLQI (SD) T0	14.63 (7.14)	15.76 (6.88)	0.401		14.26 (7.35)	15.51 (6.79)	0.38		14.79 (7.7)	14.97 (6.67)	0.887	
Mean POEM (SD) T0	19.53 (7.04)	22.2 (4.95)	0.061	**OR 0.93 (0.88–0.98), *p* = 0.006**	19.47 (7.29)	20.86 (6)	0.344		19.95 (7.45)	20.34 (6.14)	0.911	
Mean NRSp (SD) T0	8.31 (1.91)	8.53 (1.77)	0.619		8.35 (1.89)	8.37 (1.87(	0.998		8.38 (1.76)	8.35 (1.96)	0.884	

## Data Availability

The raw data supporting the conclusions of this article will be made available by the authors on request.

## Abbreviations

AD	atopic dermatitis
SCORAD	scoring atopic dermatitis
EASI	eczema area severity index
DLQI	dermatology life quality index
POEM	patient-oriented eczema measure
NRSp	numerical rating scale pruritus
ER	early responder
SR	super responder
LR	long responder
N	number
SD	standard deviation
OR	odds ratio
M	male
*p*	*p*-value
